# Maintenance of Positive Diversity-Stability Relations along a Gradient of Environmental Stress

**DOI:** 10.1371/journal.pone.0010378

**Published:** 2010-04-27

**Authors:** Tamara N. Romanuk, Richard J. Vogt, Angela Young, Constance Tuck, Mather W. Carscallen

**Affiliations:** 1 Department of Biology, Dalhousie University, Halifax, Nova Scotia, Canada; 2 Département des sciences biologiques, Université du Québec à Montréal, Montréal, Quebec, Canada; University of Sheffield, United Kingdom

## Abstract

**Background:**

Environmental stress is widely considered to be an important factor in regulating whether changes in diversity will affect the functioning and stability of ecological communities.

**Methodology/Principal Findings:**

We investigated the effects of a major environmental stressor (a decrease in water volume) on diversity-abundance and diversity-stability relations in laboratory microcosms composed of temperate multi-trophic rock pool communities to identify differences in community and functional group responses to increasing functional group richness along a gradient of environmental stress (low, medium, and high water volume). When a greater number of functional groups were present, communities were less temporally variable and achieved higher abundances. The stabilizing effect of increased functional group richness was observed regardless of the level of environmental stress the community was subjected too. Despite the strong consistent stabilizing effect of increased functional group richness on abundance, the way that individual functional groups were affected by functional group richness differed along the stress gradient. Under low stress, communities with more functional groups present were more productive and showed evidence of strong facilitative interactions. As stress increased, the positive effect of functional group richness on community abundance was no longer observed and compensatory responses became more common. Responses of individual functional groups to functional group richness became increasing heterogeneous are stress increased, prompting shifts from linear diversity-variability/abundance relations under low stress to a mix of linear and non-linear responses under medium and high stress. The strength of relations between functional group richness and both the abundances and temporal variability of functional groups also increased as stress increased.

**Conclusions/Significance:**

While stress did not affect the relation between functional group richness and stability *per se*, the way in which functional groups responded to changes in functional group richness differed as stress increased. These differences, which include increases in the heterogeneity of responses of individual functional groups, increases in compensatory dynamics, and increases in the strength of richness-abundance and richness-variability relations, may be critical to maintaining stability under increasingly stressful environmental conditions.

## Introduction

Developing an understanding of the potential consequences of environmental change and biodiversity loss is one of the most important challenges currently facing ecologists [1], [2]. While many of the consequences of declines in diversity for the continued functioning and stability of ecosystems are well-documented [3]–[6], the mechanisms underlying how diversity regulates stability and how environmental drivers will interact with species-loss to affect the stability of ecological systems are less well understood [1].

Environmental stress is widely considered to be an important factor regulating whether changes in diversity will affect the functioning and stability of ecological communities. Despite considerable work on how sources of stress and other environmental filters affect the diversity of local communities [7]–[9], relatively fewer studies have investigated how sources of environmental stress impact diversity-ecosystem functioning relations. Mulder et al. [10] reported a positive effect of diversity on productivity in moss and liverwort communities exposed to experimental drought but found no effect of diversity under constant conditions. In contrast, Pfisterer and Schmid [11] found that a drought perturbation weakened the effect of diversity on plant community biomass. Similarly, in algal microcosms, Zhang and Zhang [12] reported that a cold perturbation weakened positive diversity effects. While the results of studies that manipulate both diversity and environmental stress generally conclude that stress modulates the strength of diversity effects, the direction of the effect appears idiosyncratic, with environmental stress heightening [10] or weakening [11], [12] biotic effects depending on the study.

One potential explanation for this disparity in results is that the effect of environmental stress may differ along a gradient of severity. It is also likely that the effect of environmental stress will differ based on the stressor, the focal community, and whether the community is adapted to the type of stress imposed. Moderate environmental stress has been shown to lead to increased facilitation between species, thus heightening the role of biotic interactions, contributing to diversity effects that are otherwise not observed under more constant conditions [10]. In contrast, high levels of environmental stress might cancel positive biotic effects as species begin to respond similarly to environmental conditions [12], [13]. These divergent predictions underscore the need for more experimental studies that manipulate diversity and stress along a gradient of severity. Such an approach could also help identify mechanisms by which ecosystem functions are maintained under increasingly stressful environmental conditions.

In ephemeral freshwater rock pools, desiccation is the dominant environmental driver, killing organisms directly and exerting a strong effect on species composition, even though these organisms are well adapted to desiccation stress [14], [15]. We manipulated the number of functional groups of zooplankton meiofauna in temperate multi-trophic rock pool communities in laboratory microcosms to determine whether a press perturbation (a decrease in water volume) would alter the effect of increases in functional group richness (FGR) on: 1) total community abundance (AB_C_) and within-functional group abundance (AB_FG_) as well as 2) temporal variability in total community abundance (CV_C_), temporal variability of within-functional group abundance (CV_FG_) and mean temporal variability across functional groups (mean CV_FG_).

## Methods

Rock pool water from rain-fed pools located in the supralittoral zone was collected at Prospect Bay, Nova Scotia (44′ 28.112N, 63′ 47.663W) in August 2007. Rock pools in this region are found along exposed coastlines and form in granitic depressions. The pools range in size from ∼20 cm to ∼3 meters in diameter and contain a diverse suite of microbes, phytoplankton, and meiofauna including cladocerans, ostracods, copepods, nematodes, and insect larvae. We used functional groups rather than the number of species as our measure of diversity as it is not simply the number of taxonomic species that is important for ecosystem functioning but the diversity of ecological roles that are present in a community [16]–[20]. Using functional groups, as opposed to taxonomic species, is common in biodiversity-ecosystem functioning studies that have been conducted in plant communities [16], [21]–[23] but relatively few studies have applied this approach to multi-trophic communities. Rock pool meiofauna differ strongly in functional traits related to feeding [24]. Rock pool species were classified into functional groups based on their trophic role (e.g. predator, omnivore, herbivore), prey type (e.g. detritus, bacteria, phytoplankton) and feeding style (e.g. raptorial, grazing, filtration; Table 1) based on Barnett et al. [20]. For the species used in the experiment, the trait based functional groups correspond to the following taxonomic classifications: 1) Cyclopoida, 2) Harpacticoida, 3) Ostracoda, 4) Chydoridae (primarily *Alona and Alonella* spp.), 5) Daphniidae (*Daphnia* spp.), 6) Nematoda, and 7) Rotifera (Table 1). Identifications when counting samples were only made to the level of functional group, as described above, and not to species level.

**Table 1:** Trophic group, feeding type, and prey of the seven functional groups studied following Romanuk *et al.* [27] and Barnett *et al.* [20].

**Functional Group**	Cyclopoida	Harpacticoida	Ostracoda	Chydoridae	Daphniidae	Nematoda	Rotifera
**Trophic Group**	Omnivore-Carnivore	Omnivore-Carnivore	Omnivore-Carnivore	Herbivore-Detritivore	Herbivore	Omnivore	Omnivore
**Feeding Type**	Raptorial	Grazing	Grazing	Filtration	Filtration	Grazers	Filtration
**Prey**	Ciliates and Nauplii	Benthic Detritus	Detritus, Zooplankton, Rotifers	Bacteria and Benthic Diatoms	Bacteria and Phytoplankton	Detritus and Bacteria	Detritus, Bacteria, Phytoplankton

To establish the initial gradient of functional group richness we used a dilution series (100%, no dilution; 75%, diluted with 25% filtered rock pool water; 50%, diluted with 50% filtered rock pool water; 25%, diluted with 75% rock pool water). Rock pool water was filtered through a 63 µm mesh Nitex net, which removed all adult meiofauna but not the associated microbial community, phytoplankton, or particles of detritus smaller than 63 µm. Dilution series have been shown to successfully manipulate the diversity of various cultures including bacteria [25] and rock pool meiofauna [26], [27], [28]. After dilution, the microcosms were allowed a period of re-growth to ensure that the functional group manipulation was not confounded by differences in density [26]. This method was effective in establishing microcosms that varied in functional group richness (see Supplementary Data; Fig. S1), but the dilution series manipulation would also have affected species richness within functional groups, a variable that we did not control for. Previous studies in tropical rock pools have shown that two weeks is typically long enough for re-growth to occur to similar levels of abundance due to the fast generation times of the organisms which typically vary from days to one to two weeks [28]. This method of manipulating diversity leads to a gradient of functional group richness that over time does not always correspond to the initial dilution series manipulation (see Supplementary Data; Fig. S1).

Thirty-six communities were housed in the laboratory at 22°C on an approximate 12 hour day/night cycle in clear rectangular plastic microcosms (20 cm×10 cm×10 cm, maximum volume 1.5 L) with 500 ml (low stress), 250 ml (medium stress), or 100 ml (high stress) of water. The experiment ran for a total of 12 weeks and live counts were performed at week 2, 4, 6, 8, and 10. Based on the range in generation times for the species in this system 12 weeks is long enough to ensure that our results are not dominated by transient dynamics. To perform the live counts, 10% of the water was removed from each microcosm after stirring to homogenize the contents and all individuals were counted and assigned to their functional groups. Visual observation of entire microcosms was also used to determine the presence of functional groups at very low abundances. When a functional group was observed in the microcosm, but was not taken in the sub-sample (28 out of 180 samples), it was included in the calculation of mean functional group richness but was omitted from abundance estimates. Throughout the experiment water volume was maintained according to prescribed treatments by re-filling the microcosms with rock pool water that had been filtered through a 63 µm Nitex net.

### Statistical Analyses

#### Data analysis

All calculations were done using counts from only the last three sampling dates to ensure that 1) the estimate of CV was not affected by initial rapid changes in population growth/decline that occur when the microcosms are initially assembled and 2) that abundance estimates are not confounded by the dilution manipulation which initially affects abundance (Supplementary information: Fig. S1). Temporal variability in abundance was calculated as coefficients of variation (CV; standard deviation/mean), which standardize for differences in abundance [5]. Community variability (CV_C_) was calculated as the CV of the total abundance of all individuals in each replicate. Mean functional group variability (CV_mean_) was calculated as the mean of each functional group's CV in a microcosm:
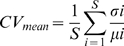
where CV_mean_ is the mean functional group (FG) variability of all FGs present in a microcosm, S is the number of functional groups, σ_i_ is the standard deviation of abundance of FG *i* during the course of the three censuses, and μ_i_ is the mean abundance of each functional group *i* over the same censuses. This method yields a single measure of functional variability per replicate and can be used to relate community (CV_C_) and functional group variability (CV_mean_) directly [28]–[30]. Variability of each functional group (CV_FG_) was also calculated.

#### Effects of environmental stress and functional group richness on abundance and temporal variability

We used general linear models (GLM) to test for interactions between environmental stress (low stress, medium stress, high stress) and mean functional group richness (FGR) on total community abundance (AB_C_) and total abundance within each functional group (AB_FG_), as well as on temporal variability in total community abundance (CV_C_), temporal variability in abundance for each functional group (CV_FG_), and mean temporal variability across the FGs CV_FGS_ (mean CV_FG_). To fit the GLMs we first used a ‘homogeneity of slopes model’ to test for significant differences in slopes based on environmental stress. Where significant differences in slopes were found a ‘separate slopes’ model was used in further analysis (e.g. AB_C_ in Table 2); where no significant differences in slopes were found, ANCOVA was used in further analysis (e.g. CV_C_ in Table 2). A separate slopes model is used in the former case, as a traditional analysis of covariance (ANCOVA) is inappropriate when the categorical and continuous predictors interact in influencing responses on the outcome [31].

**Table 2:** GLM results for effect of mean functional group richness (FGR) and environmental stress (ENV) on community variability (CV_C_), mean functional group variability (CV_mean_), and total community abundance (AB_C_).

Community variability (CV_C_)					
	SS	df	MS	F	p
Intercept	1.462	1	1.462	21.327	>0.001
FGR	0.463	1	0.463	6.753	0.014
ENV	0.018	2	0.009	0.129	0.879
Error	2.194	32	0.069		

Mean functional group variability (CV_mean_)					
	SS	df	MS	F	p
Intercept	2.167	1	2.167	104.744	>0.001
FGR	0.325	1	0.325	15.690	0.001
ENV	0.093	2	0.046	2.246	0.126
Error	0.538	26	0.021		

Total community abundance (AB_C_)					
	SS	df	MS	F	p
Intercept	209486.100	1	209486.100	2.013	0.166
ENV*FGR	1517994.516	3	505998.172	4.861	0.007
Treatment	500307.045	2	250153.523	2.403	0.108
Error	3122688.817	30	104089.627		

#### Mechanisms

We explored two mechanisms thought to underlie diversity-abundance and diversity-stability relationships: compensation and facilitation. Compensation is said to have occurred when the abundances of a species or functional group increase in order to balance a decrease in the abundances of other species or other functional groups, such that overall community abundances remain constant. In the case of facilitation, on the other hand, increases in abundance are a direct consequence of increases in the abundances of other species due to positive interactions. To detect evidence for compensation or facilitation we computed variance ratios, VR, as the ratio of the temporal variance of total community abundance to the sum of the variances in abundance of the functional groups [32]–[34]. For this test a ratio less than 1 is indicative of compensation and a ratio greater than 1 could be construed as potential evidence for facilitation.

To determine whether the effects of FGR on AB_FG_ and CV_FG_ of each functional group were modulated by stress and to identify whether functional groups might respond differently to FGR as stress increased we plotted total abundances within functional groups (AB_FG_) and functional group variability (CV_FG_) for each functional group as a function of FGR and fit linear and second-order polynomial models to the relationship within each stress treatment. An F-test was used to determine significant differences between linear and second-order terms when both were significant. The relationship was visually inspected to categorize the pattern as either hump-shaped or u-shaped. We used a significance level of p = 0.1 because many of these trends may be biologically important but difficult to detect at p = 0.05 due to low sample sizes. All analyses were conducted in Statistica v.6.0 [31].

## Results

### Temporal trends in functional group richness and abundance

Community functional group richness (FGR) ranged from one (cylopoid copepods) to seven (the maximum potential FGR; mean 3.8±1.21 SD). FGR increased from a mean value of 3.05 in the second week through to a mean of 4.78 during the sixth week before declining again to a mean value of 3.08 by the tenth week (no significant difference between week 2 and 10, Tukey post-hoc test p = 0.658; Supplementary information: Fig. S1). The increases in FGR from the first sampling date (week 2) to the second sampling date (week 4) were driven by the presence of harpacticoid and nematode eggs as well as resistant cysts of rotifers that were not observed until the second sampling period (week 4). The decrease in FGR toward the end of the experiment occurred following a number of local extinctions, primarily of *Daphnia* spp. and chydorids which occurred in 28 of 36 and 23 of 36 microcosms respectively. Even though initial abundances were highest in the undiluted microcosms, by the fourth week there were only significant differences in abundance between the highest dilution level (75% dilution) and the other three dilution levels (50%, 25% and no dilution). By the eighth week there were no differences in abundance according to the initial dilution level (Supplementary Information; Fig. S1). These changes in abundance did not occur through re-growth as have previously been reported in other systems [26]–[28] but occurred instead as abundances declined over time likely due to the carrying capacity of the microcosms.

### Effects of environmental stress on community composition and functional group richness

Mean FGR was highest in the medium stress treatment (mean = 4.42,±0.59 SD; F_2,33_ = 8.477, p = 0.001). There was no significant difference in mean FGR between the low (mean = 3.97, ±0.64 SD) and high stress treatments (mean = 3.36, ±0.66 SD; Tukey HSD p = 0.059). Differences in community composition among the microcosms became more pronounced as time progressed (Supplementary Information; Fig. S2). Differences in community composition among the low and medium stress microcosms remained relatively low throughout the course of the experiment (Supplementary Information; Fig. S2). In the high stress treatments composition differed strongly among microcosms, with five microcosms losing all but the cylopoid FG, with the majority dominated by either a cyclopoid-harpacticoid community (n = 3) or an cyclopoid-ostracod (n = 2) community. In the high stress treatment only two microcosms retained more than two species by week 10.

### Effects of functional group richness and environmental stress on abundance

**Figure 1:**
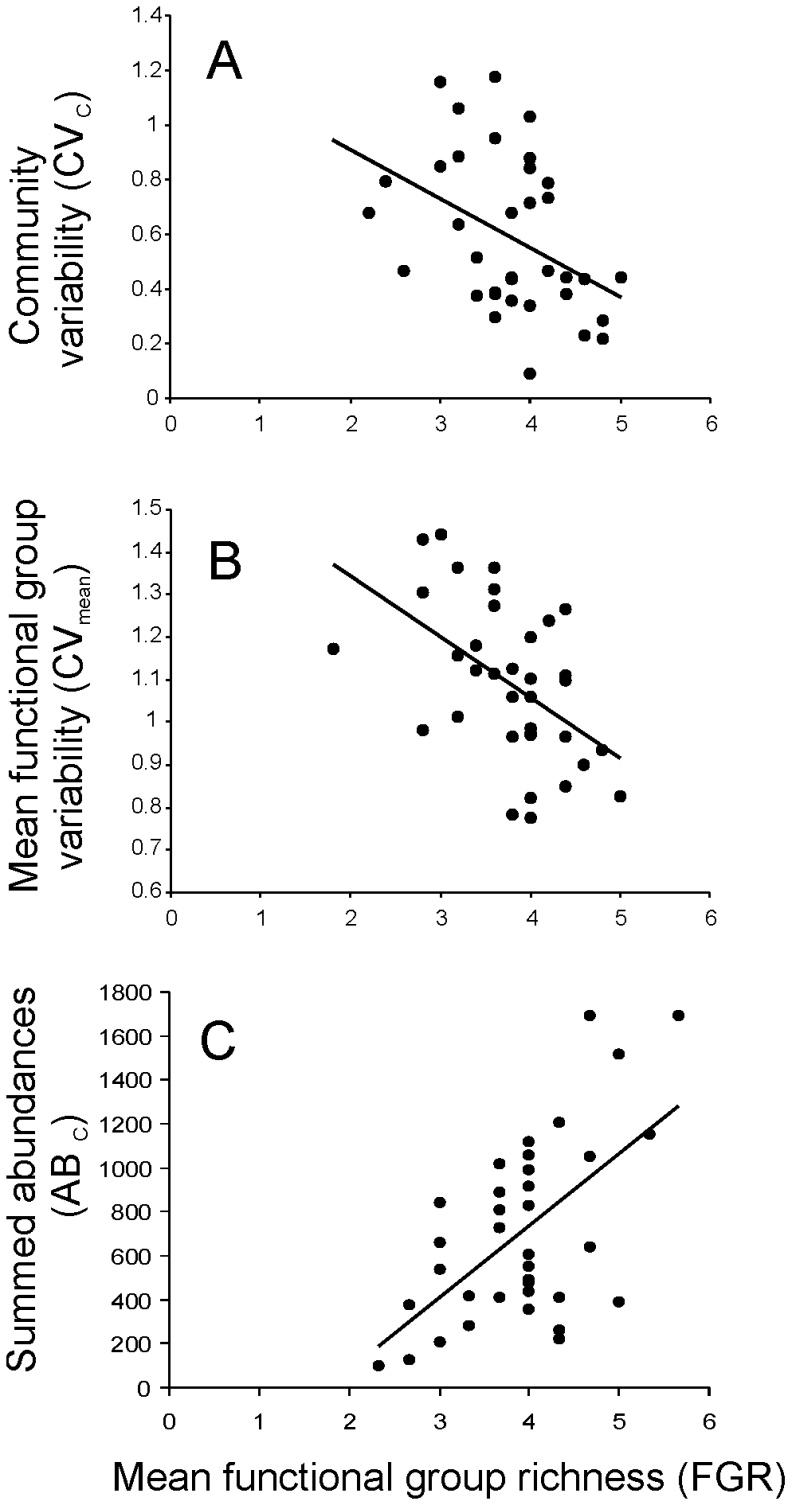
Functional group richness, temporal variability and abundance. Relationship between mean functional group richness (FGR) and A) temporal variability in total community abundance (CV_C_), B) mean temporal variability across the seven functional groups (CV_mean_), and C) total community abundance (AB_C_).

Mean AB_FG_ was lowest in the high stress treatment (mean = 139, ±101 SD; F_2,33_ = 5.364, p = 0.01). There was no significant difference in mean AB_FG_ between the low (mean = 280, ±86 SD) and medium stress treatments (mean = 288, ±171 SD; Tukey HSD p = 0.986). There was a significant interaction between stress and FGR on total community abundances (AB_C_; F_3,30_ = 4.86, p = 0.007; Table 2, Fig. 1c) with greater FGR leading to higher AB_C_ under low stress but not under medium or high stress. Across treatments, total abundance within FGs (AB_FG_) was positively correlated with FGR for five of the seven FGs (Table 3). No significant negative correlations were observed between AB_FG_ and FGR for any FG. Stress had a direct effect on AB_FG_ for chydorids, rotifers, cladocerans, ostracods, and nematodes. The only FG for which there was a significant interaction between FGR, AB_FG_ and stress was for nematodes (F_3,30_ = 9.03, p>0.001; Table 3), with AB_FG_ increasing with FGR under medium stress but not under low or high stress.

**Table 3:** GLM results for effect of mean functional group richness (FGR) and environmental stress (ENV) on functional group abundance (AB_FG_) and functional group variability (CV_FG_) for each functional group.

	A) Mean functional group abundance (AB_FG_)			B) Functional group variability (CV_FG_)		
Functional Group		F	p		F	p
**Ostracoda**	Intercept	3.245	0.081	Intercept	15.671	>0.001
	FGR	11.678	0.002	FGR	3.845	0.059
	ENV	3.686	0.036	ENV	0.825	0.447
**Copepoda**	Intercept	0.001	0.976	Intercept	10.212	0.003
**(Cyclopoida)**	FGR	2.265	0.142	FGR	2.846	0.102
	ENV	1.635	0.211	ENV	1.535	0.231
**Chydoridae**	Intercept	0.644	0.428	Intercept	36.283	>0.001
	FGR	0.077	0.783	FGR	6.118	0.020
	ENV	6.026	0.006	ENV	4.387	0.022
**Nematoda**	Intercept	11.849	0.002	Intercept	5.434	0.026
	FGR*ENV	9.027	>0.001	FGR	8.217	0.007
	Treatment	6.865	0.004	ENV	1.784	0.184
**Rotifera**	Intercept	11.405	0.002	Intercept	11.405	0.002
	FGR	19.091	>0.001	FGR	19.091	>0.001
	ENV	4.905	0.014	ENV	4.905	0.014
**Copepoda**	Intercept	2.261	0.142	Intercept	2.261	0.142
**(Harpacticoida)**	FGR	6.184	0.018	FGR	6.184	0.018
	ENV	0.064	0.938	ENV	0.064	0.938
**Daphniidae**	Intercept	1.946	0.173	Intercept	1.946	0.173
	FGR	5.306	0.028	FGR	5.306	0.028
	ENV	6.988	0.003	ENV	6.988	0.003

**Figure 2:**
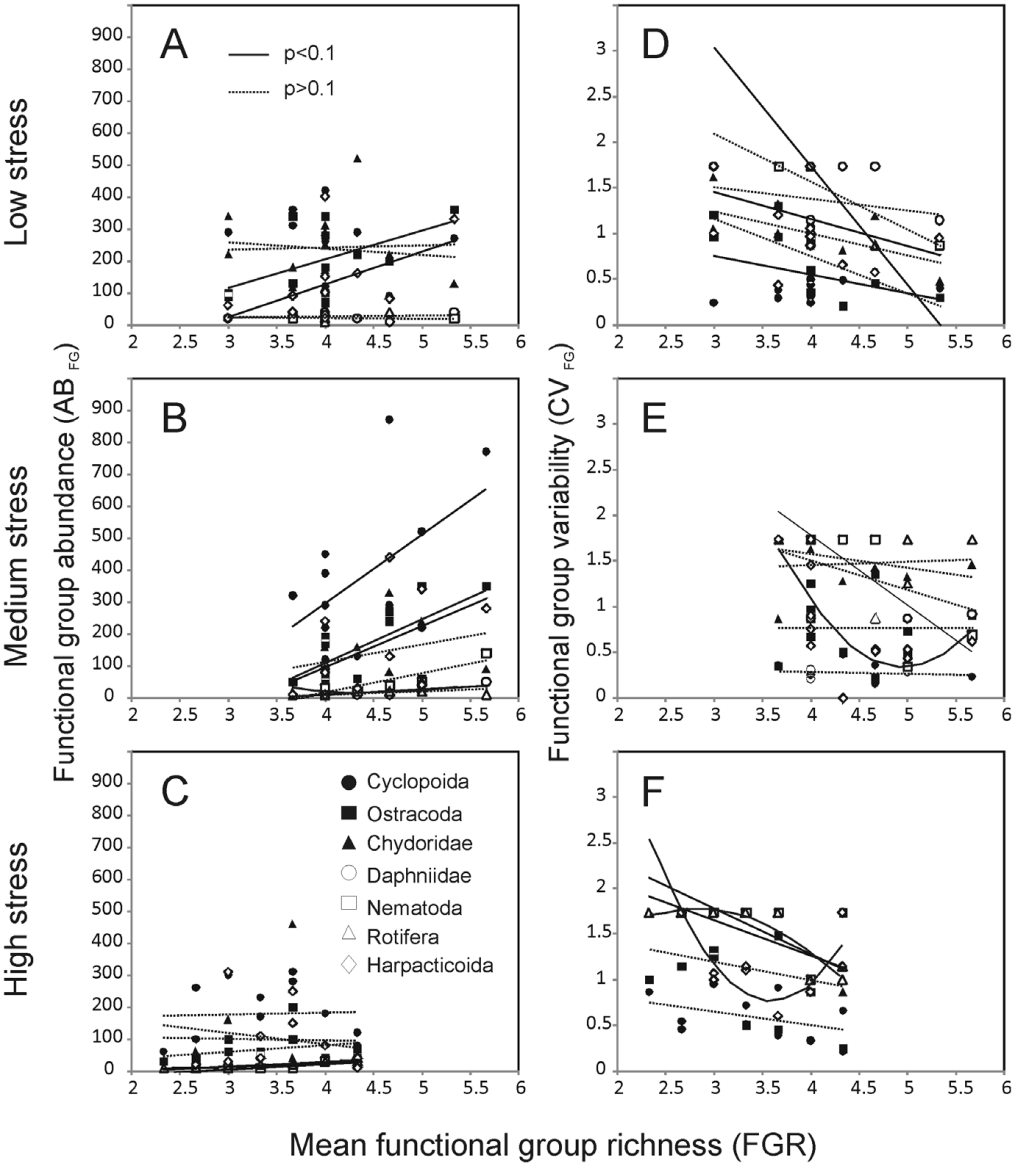
Response diversity of functional groups. Response diversity for low stress (A, D), medium stress (B, E), and high stress (C, F) treatments for mean functional group richness (FGR) and total abundance within functional groups (AB_FG_; A–C) and functional group variability (CV_FG_; D–F).

The specific form of the FGR-AB_FG_ relationships shifted from positive linear responses under low (all linear) stress to a mix between linear and u-shaped relations under medium and high stress (Fig. 2a–c, Table 4). The average explained variance was greater under medium (44.6%) and high (39.8%) stress than under low stress (26.1%).

**Table 4:** Relations between mean functional group richness (FGR) on A) functional group abundance (AB_FG_) and B) functional group variability (CV_FG_) under low, medium, and high stress conditions showing number of replicates that contained the functional group in each treatment (n), r^2^, p, and the best fit* (linear or curvilinear (hump-shaped or u-shaped)) for each regression.

		A) Functional group abundance (AB_FG_)			B) Functional group variability (CV_FG_)		
Stress Treatment	*n*	r^2^	p	Fit*	r^2^	p	Fit*
**Low stress**							
Ostracoda	12	0.148	0.217		0.536	0.007	linear
Cyclopoida	12	<0.001	0.990		0.092	0.337	
Chydoridae	12	0.011	0.741		0.249	0.098	linear
Nematoda	2						
Rotifera	5	0.889	0.016	linear	1	<0.001	linear
Harpacticoida	12	0.491	0.011	linear	0.143	0.225	
Daphniidae	10	0.028	0.647		0.037	0.593	
Mean		0.261	sig = 2		0.317	sig = 3	
**Medium stress**							
Ostracoda	12	0.486	0.012	linear	<0.001	0.995	
Cyclopoida	12	0.286	0.073	linear	0.023	0.634	
Chydoridae	11	0.093	0.363		0.005	0.995	
Nematoda	7	0.810	0.006	linear	0.657	0.027	linear
Rotifera	9	0.160	0.285		0.059	0.525	
Harpacticoida	12	0.304	0.063	linear	0.549	0.028	u-shaped
Daphniidae	6	0.981	0.001	u-shaped	0.197	0.378	
Mean		0.413	sig = 5		0.364	sig = 2	
**High stress**							
Ostracoda	12	0.098	0.321		0.063	0.431	
Cyclopoida	12	0.069	0.410		0.184	0.188	linear
Chydoridae	8	<0.001	0.967		0.688	0.011	linear
Nematoda	5	0.919	0.081	u-shaped	0.919	0.081	hump-shaped
Rotifera	8	0.869	<0.001	linear	0.677	0.012	linear
Harpacticoida	10	0.034	0.610		0.679	0.019	u-shaped
Daphniidae	1						
Mean		0.481	sig = 2		0.605	sig = 4	

Also shown is the mean r^2^ within each treatment and number of significant fits.

**Figure 3:**
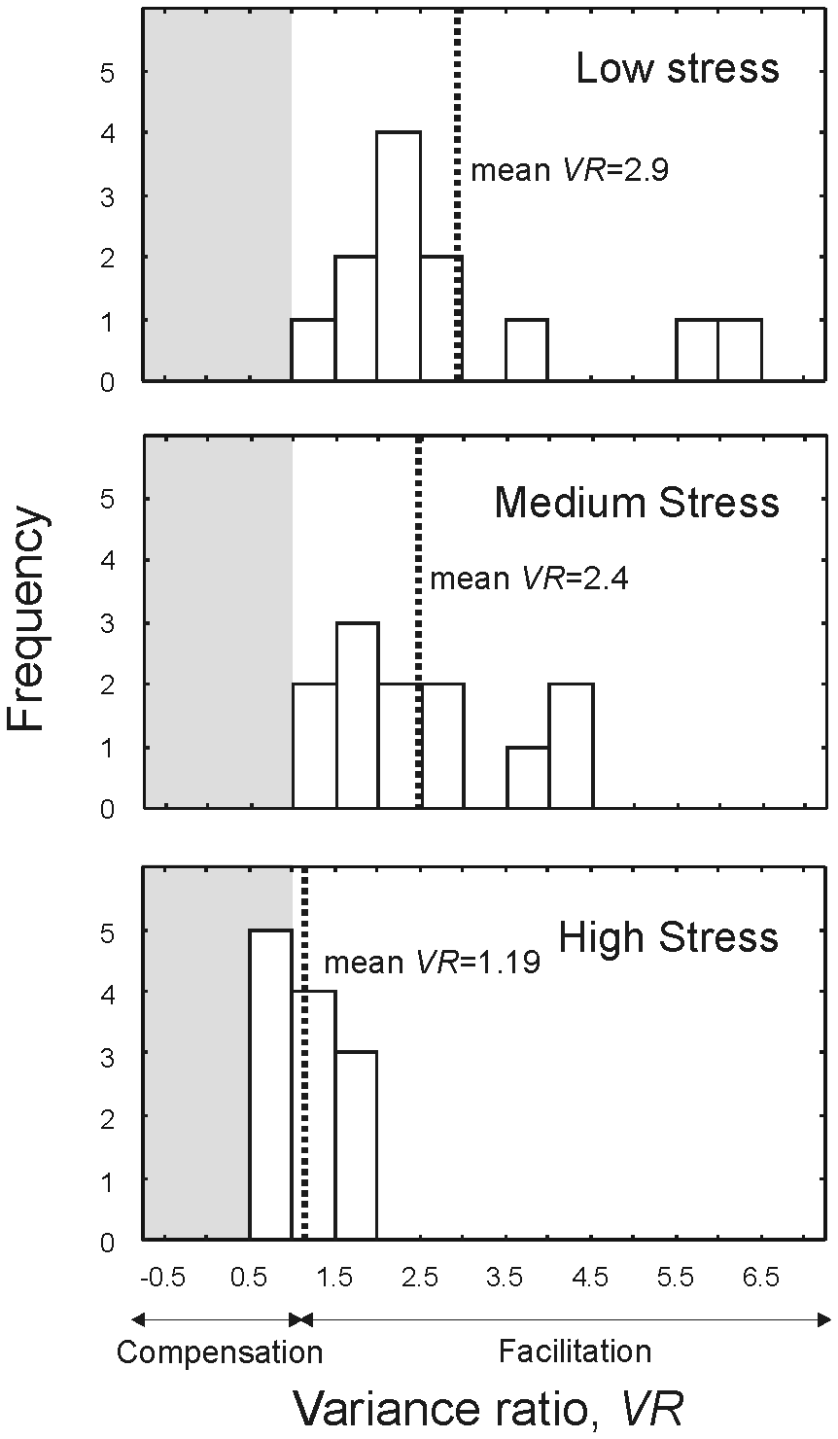
Variance ratios, *VR*, across environmental stress treatments. Histograms showing frequency of variance ratios, *VR*, for functional group abundance in low stress (A), medium stress (B), and high stress (C) treatments. Values greater than 1 reflect positive interactions and values less than 1, which are shaded in grey, reflect negative interactions. Dotted lines show the mean variance ratio, mean *VR*, for each treatment.

*Fit was determined by fitting a linear model followed by a second order polynomial model. If the linear model was not significant and the polynomial model was significant the polynomial model is shown. If both models were significant we used an F-test to determine the model that best fit the data. Only models with a p-value less than 0.2 are listed as linear, hump-shaped, or u-shaped in the table. A significance level of p = 0.1 was used (see Fig. 3 for trends).

### Effects of functional group richness and environmental stress on temporal variability

Across all stress treatments, temporal variability in total community abundance (CV_C_) decreased with increasing FGR (F_1,32_ = 6.73, p = 0.014; Fig. 1a, Table 2). CV_C_ was unaffected by stress (F_2,32_ = 0.13, p = 0.879) and there was no significant interaction effect between stress and FGR on CV_C_ (F_2,30_ = 5.67, p = 0.573). A similar result was observed for temporal variability in mean functional group abundance (CV_mean_). Across all treatments CV_mean_ decreased with increasing FGR (F_1,30_ = 15.69, p>0.001; Fig. 1b, Table 2). CV_mean_ was unaffected by stress (F_2,32_ = 2.24, p = 0.126) and there was no significant interaction effect between stress and FGR on CV_mean_(F_2,30_ = 0.63, p = 0.539).

Within functional groups, CV_FG_ declined with increasing FGR for all FGs except for cyclopoid copepods (p = 0.102; Table 3) and ostracods, however the latter relation was only marginally insignificant (p = 0.059). Stress had a significant destabilizing effect on CV_FG_ only for chydorids (F_2,27_ = 4.39, p = 0.022). There was no interaction between FGR and stress for any FG (Table 3). The statistically significant effect of stress on CV_FG_ for *Daphnia* spp. and rotifers (Table 3) was driven by the near absence of *Daphnia* spp. in the high stress treatment (only 1 high-stress microcosm contained *Daphnia* spp.) and the near absence of rotifers in the low stress treatment (only 2 microcosms contained rotifers) by the end of the experiment.

Despite the absence or only marginal effect of stress identified by the GLMs, FGR-CV_FG_ relationships were strongly affected by stress (Fig. 2d–f). In the low stress treatment CV_FG_ declined linearly (n = 3) with FGR and for all seven FGs the trend was towards a decline in CV with increasing FGR. As stress increased, the responses of the different FGs to increasing FGR became increasingly heterogeneous and symmetric. In the medium stress treatment one response was u-shaped and one showed a linear decline in CV_FG_ with increasing FGR. Unlike the trends towards a decline in CV_FG_ with increasing FGR for all seven FGs observed under low stress, under medium stress CV_FG_ of ostracods and cyclopoid copepods showing no response to FGR. Under high stress CV_FG_ responses to increasing FGR were hump-shaped for nematodes, u-shaped for harpacticoid copepods, and declined linearly with FGR for chydorids, cyclopoid copepods, and rotifers. Interestingly, none of the FGR-CV_FG_ responses were consistent among all three treatments. For example, CV_FG_ for harpacticoid copepods was independent of FGR under low stress and u-shaped under medium and high stress. The average explained variance was greater under high stress (53.5%) than in the low (33.4%) or medium stress treatments (21.3%).

### Compensation and Facilitation

In the control and medium stress treatments, the variance ratios, *VR*, were all greater than 1 which can be interpreted as evidence of facilitative dynamics (Fig. 3). In contrast, under high stress 5 of 12 *VRs* were less than 1, suggestive of compensatory dynamics. Mean *VR* decreased as environmental stress increased from 2.9 (1.56 SD) in the control, to 2.4 (1.13 SD) under medium stress, to 1.19 (0.36 SD) under high stress suggesting that the strength of interspecific interactions decreased as stress increased (F_2,33_ = 7.41, p = 0.002).

## Discussion

This study demonstrates that rock pool invertebrate communities with higher functional group richness are more temporally stable, an effect that is independent of exposure to environmental stress. This stabilizing effect of functional group richness was observed when stability was calculated as temporal variability of aggregated community abundances (Fig. 1a) as well when temporal variability in abundance was averaged across functional groups (Fig. 1b). Relations between functional group richness and the temporal variability in abundances of individual functional groups, however, were differentially affected by exposure to environmental stress. Within the low stress treatment, all functional groups exhibited a trend towards stability increasing as the number of functional groups increased (Fig. 3d–f). As exposure to stress increased, however, relations between the stability of individual functional groups and functional group richness became more variable, with some demonstrating more neutral or non-linear relations. In order to reconcile the consistent effect of functional group richness on community stability with the more variable relations at the level of individual functional groups as exposure to stress increased, we investigated the underlying mechanistic basis by which functional group richness conferred stability at the community level. Our results suggest that the frequency and strength of facilitative interactions was higher under low stress and that compensatory dynamics became more common as stress increased (Fig. 3).

As noted above, we observed strong decreases in community variability and mean functional group variability with increasing functional group richness irrespective of exposure to environmental stress (Fig. 1). A decrease in population and community variability with increasing species richness has been reported previously for tropical rock pool meiofauna [24], [26], [28], [35]–[37], pond zooplankton [30], and some plant communities [38]. Temporal variability in functional group abundance declined significantly with increasing functional group richness (FGR) for all functional groups except for cyclopoid copepods and ostracods, both of whom showed negative, albeit insignificant, trends (Table 3). Despite these strong consistent trends, functional group richness affected functional groups differently across the three environmental stress treatments, showing that there was high response diversity across the functional groups in response to both functional group richness and stress (Fig. 3).

Environmental stress had a direct effect on the abundance of several groups including chydorids, ostracods, rotifers, daphnia, and nematodes, leading to strong divergence in the composition of many communities, particularly between the low and high stress treatments. The specific form of the relation between abundances within functional groups and functional group richness was highly variable across the environmental stress treatments (Fig. 3). Under low stress, the forms of the abundance-richness relations were either linear or neutral. Under medium stress the abundance-richness relations were mostly positive and linear. Under high stress, the forms of the abundance-richness relations were generally neutral with one linear and one u-shaped relation observed.

It has previously been suggested that increasing environmental stress may cancel or weaken positive effects of diversity as species begin to respond more similarly to environmental conditions as stress increases [11], [12], [39]. We noted the opposite trend: the mean strength of the relations between functional group richness and abundance of functional groups was higher in the medium and high stress treatments than the low stress treatments. These results suggest that although stress may not affect richness-variability relations *per se* at the community-level, stress did affect the way that functional groups responded to differences in functional group richness.

An investigation of variance ratios within communities provided further evidence for differences among communities subjected to different levels of environmental stress. Variance ratios represent the temporal variance of total community abundance relative to the sum of variances in abundance of the functional groups represented [32]–[34]. Ratios less than 1 are indicative of compensatory dynamics while ratios greater than 1 can often represent facilitative interactions. Mean variance ratios decreased from 2.9 under low stress to, to 2.4 under medium stress, to 1.19 under high stress, suggesting that facilitative interactions likely contributed to community stability across the stress gradient (Fig. 3). As stress increased the mean magnitude of the positive interspecific interactions decreased. In the low stress treatment 100% of variance ratios were greater than 1. In the high stress treatment 42% of variance ratios were less than 1. These results suggest that a shift may have occurred towards more compensatory dynamics under high stress with reductions in the abundance of some species being offset by augmentations in abundance of other species.

Despite the emergence of compensatory dynamics under high stress, in general facilitative dynamics were more common across all three stress levels. In natural aquatic microcosms, facilitative interactions among detritivorous species are generally more common than competitive interactions [40]–[43]. Facilitation arises when one functional group enhances the biomass or abundance of another functional group by modifying the environment or by enhancing access to resources [44]. For example, Mulder et al. [10] found that bryophyte communities exposed to constant conditions exhibited no relation between richness and productivity, but that a positive effect between richness and biomass became apparent under drought. They attributed the positive effect of richness on biomass under these conditions to facilitative interactions between species, which actually increased the survivorship of otherwise drought intolerant species. While our results differ from those of Mulder et al. [10] in some respects, our results support the hypothesis that positive interactions may be an important and underemphasized mechanism linking high diversity to production and stability. However, our results also show that compensatory dynamics may have contributed to community-level stability as stress increased. Previous studies conducted in tropical rock pools have suggested that facilitation might arise through detrital processing chains [24]. Although we did not specifically address the means by which facilitation might be conferring community-level stability in this experiment, it is likely that facilitation among detritivores underlie some of the positive effects of increasing functional group richness on community and functional group abundances that we observed.

Taken together our results suggest that functional groups can respond differently with respect to their abundances and stability to increasing functional group richness along a gradient of environmental stress, with predominantly linear responses under low stress and increasingly heterogeneous and stronger responses as stress increases. Increases in the heterogeneity of responses of different functional groups and shifts from predominately facilitative to a mix of facilitative and compensatory dynamics may be important mechanisms by which stability is maintained under increasingly stressful environmental conditions. Further studies incorporating diversity within as well as across functional groups are needed to evaluate and compare the robustness of our results to studies that manipulate the number of species rather than the number of functional groups in a community.

## Supporting Information

Figure S1 A) Total abundance and B) number of functional groups at weeks 2, 4, 6, 8, and 10 showing means, minimum (Min) and maximum (Max) values, and standard errors (± SE) for the four levels of the dilution series (25%, 50%, 75%, and 100%).
(1.48 MB TIF)

Figure S2 Relative abundance of the seven functional groups at week 6, 8, and 10 in the low stress (n = 12), medium stress (n = 12), and high stress (n = 12) treatments. The community composition is shown for each microcosm (n = 36).
(1.59 MB TIF)
