# Supplementary figures and images for: Maintenance of Positive Diversity-Stability Relations along a Gradient of Environmental Stress
Source: PLoS One. 2010 Apr 27;5(4):e10378. doi: 10.1371/journal.pone.0010378 (PMC2860506; doi:10.1371/journal.pone.0010378)

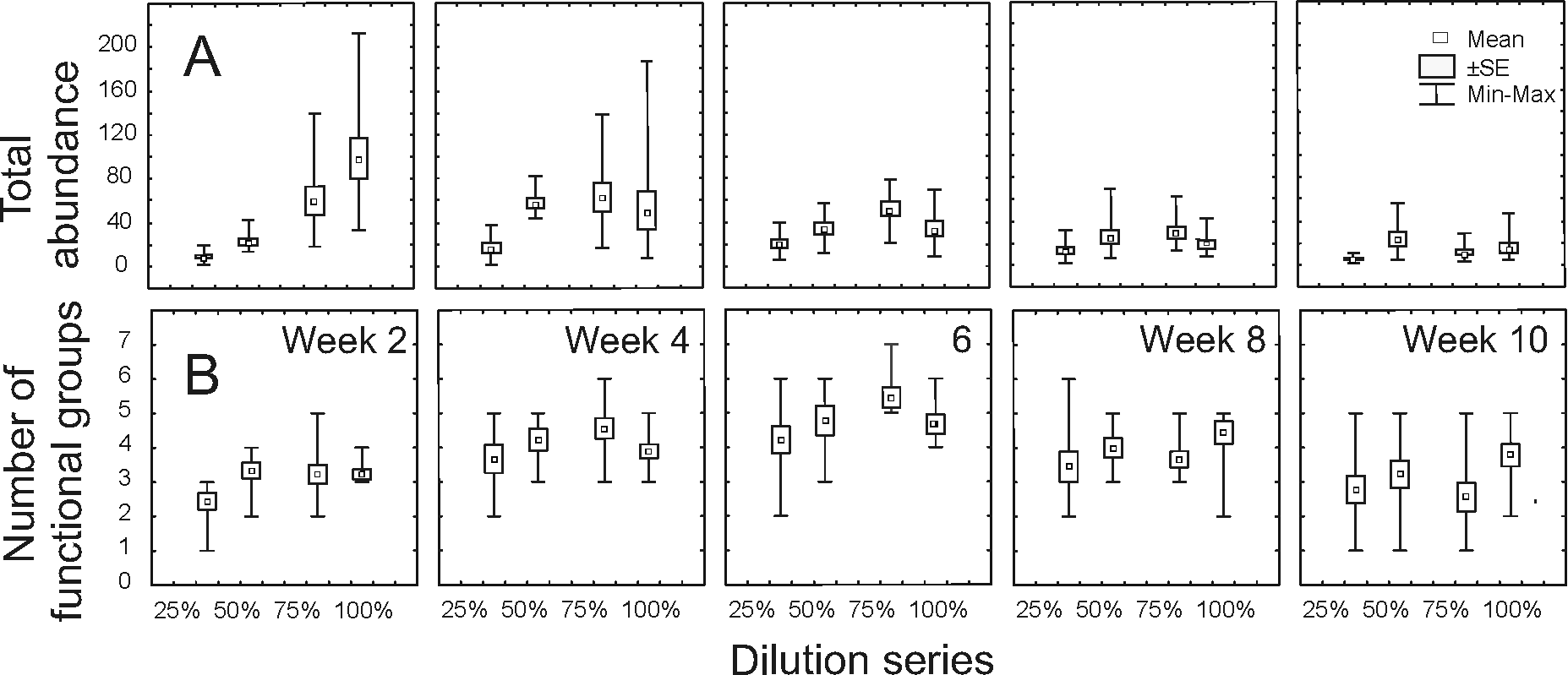

Supplement: Figure S1 — A) Total abundance and B) number of functional groups at weeks 2, 4, 6, 8, and 10 showing means, minimum (Min) and maximum (Max) values, and standard errors (± SE) for the four levels of the dilution series (25%, 50%, 75%, and 100%). (1.48 MB TIF) [file pone.0010378.s001.tif]
